# Efficacy of different allergen-specific immunotherapies for the treatment of allergic rhinitis in children and adults: an umbrella review

**DOI:** 10.3389/fimmu.2025.1658826

**Published:** 2025-09-25

**Authors:** Yu He, Xinghong Liu, Bin Zhou, Tianrong Dai

**Affiliations:** ^1^ School of Traditional Chinese Medicine, Chongqing University of Chinese Medicine, Chongqing, China; ^2^ School of Clinical Medicine, Chengdu University of Traditional Chinese Medicine, Chengdu, China; ^3^ Otolaryngology Department, Ya’an People’s Hospital, Ya’an, China

**Keywords:** allergic rhinitis, allergen immunotherapy, efficacy, meta-analysis, systematic review, umbrella assessment

## Abstract

**Objective:**

The aim of this study was to summarize the therapeutic efficacy of various allergen immunotherapy (AIT) in the treatment of allergic rhinitis (AR) among different populations and for different allergens.

**Methods:**

Systematic reviews or meta-analyses related to the efficacy of AIT in the treatment of AR until October 2024 were retrieved from PubMed, Web of Science, Embase, and Cochrane Library. Each study was independently evaluated by two investigators in accordance with the inclusion and exclusion criteria. The methodological quality was assessed using AMSTAR 2, and the quality of evidence was evaluated by the GRADE system.

**Results:**

A total of 16 SRs/Mas were included. The methodological quality was evaluated by AMSTAR 2, with 5 rated as “low” and the remainder as “very low”. The quality of the evidence was assessed using the GRADE system. It was found that the quality of evidence in most studies was unsatisfactory. Among the included articles, six had moderate-quality evidence, six had low-quality evidence, and four had very low-quality evidence, with no high-quality evidence. There was a moderate degree of overlap among the included literature. After conducting an overall efficacy evaluation of the extracted data, it was found that SLIT, SCIT, and LNIT were effective in the treatment of AR, while cluster SCIT and ILIT had no significant efficacy compared with placebo.

**Conclusion:**

SLIT and SCIT are active and effective treatments for AR, and show significant efficacy in adults, children, and for different allergens. There are still relatively few meta-analyses and systematic reviews of cluster SCIT, ILIT, and LNIT, and there is still scope for further improvement in the assessment of their efficacy. Considering that the methodological quality and evidence of the systematic reviews and meta-analyses included in this study are generally low, more high-quality, large-scale, multicenter, randomized controlled clinical trials are indispensable in the future to firmly verify the efficacy of various AIT in the treatment of AR in different populations and allergens.

**Systematic review registration:**

https://www.crd.york.ac.uk/prospero/, identifier CRD42024600378.

## 1 Introduction

Allergic rhinitis (AR) is a chronic Th2-type inflammatory reaction in the nasal mucosa, triggered by allergens and mediated by specific immunoglobulin E (IgE). Common symptoms include nasal itching, sneezing, clear discharge, and congestion (1). Key allergens are seasonal pollen, dust mites, and pet dander. In the US, physician-diagnosed AR prevalence is about 15% (2), rising to 30% based on self-reported symptoms (3). In China, AR is one of six major chronic diseases, with a prevalence rate nearing 40%, notably higher among children, impacting public health significantly (4).

Treatment primarily involves intranasal and oral medications, which manage but do not cure symptoms. Allergen immunotherapy (AIT), particularly subcutaneous (SCIT), sublingual (SLIT), intradermal (IDIT), epidermal (EPIT), and intralymphatic (ILIT) therapies, targets specific allergens and is increasingly recognized as the most effective treatment. IDIT and EPIT offer advantages like shorter treatment duration and lower allergen doses (5). ILIT involves ultrasound-guided injections into subcutaneous lymph nodes (6).

Existing reviews mainly focus on the efficacy of specific allergens or treatment regimens for AR, but few have compared different AIT administration routes. This study is the first to evaluate administration routes as a key factor, comparing their effectiveness in treating AR. The results provide a basis for choosing personalized clinical approaches and fill a research gap that previously focused mostly on SLIT and SCIT. An umbrella review (UR) is a type of overarching systematic review that aims to provide reliable evidence for decision-makers when there is a growing number of existing systematic reviews (7). The purpose of this UR is to synthesize evidence from systematic reviews and meta-analyses (SRs/MAs) in order to provide high-quality evidence regarding the efficacy of various administration routes of AIT for AR, enabling their comparison.

## 2 Methods and materials

### 2.1 Protocol and registration

This study adhered to the Preferred Reporting Items for Systematic Reviews and Meta-analyses (PRISMA) (8). Additionally, it was registered with PROSPERO (CRD42024600378).

### 2.2 Search strategy

To explore the efficacy of AIT in the treatment of AR, two investigators independently conducted searches in PubMed, Web of Science, Embase, and Cochrane Library for relevant articles. The search period extended from the establishment of each database until October 2024. Search terms included: “Allergic Rhinitides,” “Allergic Rhinitis,” “Immunologic Desensitizations,” “Hyposensitization Therapies,” “Hyposensitization Therapy,” “Allergen Immunotherapy,” “Allergen Immunotherapies,” “Venom Immunotherapy,” “Venom Immunotherapies,” “Allergy Shots,” “Allergy Shot,” “Systematic Review,” “Systematic Reviews,” “meta-analysis,” “Meta-analysis,” “data pooling,” “clinical trial overview,” “clinical trial overviews,” etc. The detailed PubMed search strategy is presented in **Table 1**, and other databases were adjusted in accordance with the PubMed search strategy. Additionally, to prevent the omission of relevant meta-analyses in the initial search, references of relevant studies were reviewed.

**Table 1:** PubMed search strategy.

Query	Search term
#1	((Rhinitis, Allergic[MeSH Terms]) OR (Allergic Rhinitides[Title/Abstract])) OR (Allergic Rhinitis[Title/Abstract])
#2	(((((((((Desensitization, Immunologic[eSH Terms]) OR (Immunologic Desensitizations[Title/Abstract])) OR (Hyposensitization Therapies[Title/Abstract])) OR (Hyposensitization Therapy[Title/Abstract])) OR (Allergen Immunotherapy[Title/Abstract])) OR (Allergen Immunotherapies[Title/Abstract])) OR (Venom Immunotherapy[Title/Abstract])) OR (Venom Immunotherapies[Title/Abstract])) OR (Allergy Shots[Title/Abstract])) OR (Allergy Shot[Title/Abstract])
#3	((((((((Meta-Analysis as Topic[MeSH Terms]) OR (Meta Analysis as Topic[Title/Abstract])) OR (Clinical Trial Overviews[Title/Abstract])) OR (Clinical Trial Overview[Title/Abstract])) OR (Data Pooling[Title/Abstract])) OR (Data Poolings[Title/Abstract])) OR (Systematic Reviews[Title/Abstract])) OR (Systematic Review[Title/Abstract])) OR (Cochrane review[Title/Abstract])
#4	#1 AND #2 AND #3

### 2.3 Selection of studies

Systematic reviews and meta-analyses evaluating the efficacy of AIT in the treatment of AR were included. Each study was independently assessed by two investigators. The literature inclusion criteria were as follows: (1) Meta-analyses or systematic reviews and meta-analyses published in English; (2) The efficacy of AIT in the treatment of AR was studied; (3) Total effect sizes and 95% confidence intervals (CIs) were reported. Exclusion criteria were: (1) Repeatedly published studies; (2) The study population was non-AR patients with incomplete data or the required data could not be obtained from the original article; (3) Animal experiments, case reports, conference speeches, etc. Two investigators independently screened the literature by reading the title and abstract, and then the two investigators independently reviewed the full text to further confirm the inclusion or exclusion of relevant literature. In case of differences of opinion that could not be resolved through discussion, a third researcher determined whether to include the article.

### 2.4 Data extraction

Upon completing the data extraction form, two researchers independently extracted the following data from each systematic review and meta-analysis: the author, the country of publication, the time of publication, the number of included studies, the population of participants, the allergen, the intervention, efficacy-related outcome measures, the effect model, the effect size and 95%CI, the *p-*value or the I (2) value of the heterogeneity test. All data were independently extracted by two researchers.

### 2.5 Methodological and evidence quality evaluation

Two investigators independently assessed each including systematic review and meta-analysis. The methodological quality was evaluated using AMSTAR 2, with each item classified as “yes”, “partly yes” or “no” (9). The quality of evidence was appraised using the GRADE system, which clearly defines the quality of evidence and the strength of recommendations, and classifies the evidence into “high”, “moderate”, “low”, “very low” or “cannot be recommended” (10). The overlap of major studies included in the literature might mislead the results. To measure this overlap, the OVErviews (GROOVE) tool was employed, which computes the evidence matrix and corrected coverage area (CCA). Overlap was classified as mild if CCA<5%; If CCA≥5% and <10%, the classification was moderate. If CCA≥10% and <15%, it was classified as high. If CCA≥15%, it was classified as very high (11).

### 2.6 Strategies for data synthesis

We chose symptom scores and medication scores for AIT treatment of AR as evidence of efficacy and extracted the relevant data. The 95% confidence intervals reported in each study were utilized to assess the overall efficacy. Heterogeneity among studies was evaluated using I (2), with values greater than 50% indicating high heterogeneity. Publication bias in systematic reviews and meta-analyses was determined using the Egger test, with a *p*-value less than 0.05 indicating bias. In some studies that could not be quantitatively and comprehensively analyzed, we carried out a descriptive analysis of the outcome indicators related to the efficacy of AIT in the treatment of AR (12). To provide more detailed insights into the efficacy of AIT in subgroups with different administration routes, we conducted a subgroup analysis of data on various allergens and patient populations across each administration route.

In addition to assessing the efficacy of different administration routes of AIT in AR through symptom scores and medication scores, the impact of AIT on the progression of allergic diseases remains a critical clinical concern. This includes the development of new-onset asthma in patients with AR, as well as the progression of AR in individuals with comorbid asthma. However, due to insufficient reporting of this outcome in the original reviews included in this study, inconsistencies in its definition, and variations in the duration of follow-up, the evidence regarding new-onset asthma or the progression of AR in combination with asthma is limited. Therefore, this study presents the available findings in the form of a literature discussion, highlighting it as an important area for future research.

## 3 Results

### 3.1 Search results

**Figure 1:**
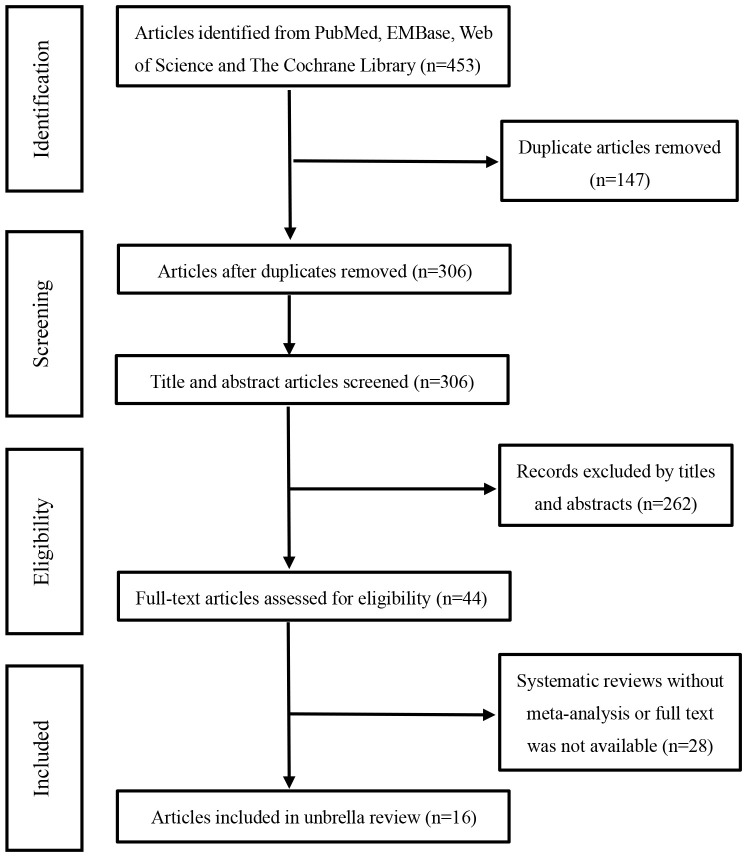
Flow chart of the literature selection.

Based on the pre-defined search strategy, we initially retrieved 453 articles. After eliminating duplicates, the number of articles was 306. By perusing titles and abstracts, 261 articles irrelevant to the selected topic were excluded. After reading the full text, an additional 29 articles were eliminated. Eventually, through the literature screening process, 16 systematic reviews and meta-analyses were incorporated into this umbrella review (13–28), as depicted in **Figure 1**. We summarized the efficacy measures of AIT in AR, using medication scores (MS) and/or symptom scores (SS) as a reference to assess the efficacy of AIT in AR, and extracted these data from the included studies.

### 3.2 Characteristics of included studies

The 16 systematic reviews and meta-analyses incorporated in this study were all published within the period from 2005 to 2023. The studies included in the analysis varied from 4 to 58, with sample sizes ranging from 134 to 3331. The AIT measures in the intervention group were also slightly different. Among them, 10 articles pertained to SLIT, 6 articles concerned SCIT, 2 articles encompassed a comprehensive study of AIT without differentiating between administration routes, and there was one related study each for cluster SCIT, ILIT, and local nasal immunotherapy (LNIT). The control group was mainly placebo. The principal characteristics of this study are elaborated in **Table 2**.

**Table 2:** Characteristics of the included studies.

Author	Year	Allergen	Number of studies	Study population	Number of treatment groups	Treatment intervention	Number of control groups	Control intervention	Outcome measures	Effect size	95%CI	*P-*value	I² value
Feng, B. H. et al. (13)	2017	HDM	25		1260	SLIT	1128	placebo	SS	SMD=-1.23	-1.74~-0.73	*p<0.001*	I²=96%
			18		963		851		MS	SMD=-1.39	-1.90~-0.88	*p<0.001*	I²=95%
			12	Children only					SS	SMD=-0.70	-1.43~0.03	*p=*0.06	
			7						MS	SMD=-1.66	-2.60~-0.71	*p=*0.006	
			10	Adults only					SS	SMD=-1.02	-1.53~-0.52	*p*<0.0001	
			6						MS	SMD=-1.31	-2.12~-0.51	*p=*0.001	
Feng, S. Y. et al. (14)	2014		4		103	cluster SCIT	77	placebo	SS	WMD=-5.91	-13.68~1.87	*p=*0.14	I²=89%
			4		103		77		MS	WMD=-1.27	-2.83~0.29	*p=0.11*	I²=94%
			2		165		163	conventional SCIT	SS	WMD = 0.16	-0.18~0.51	*p=0.36*	I²=0%
			2		165		163		MS	WMD = -0.01	-0.16~0.13	*p=0.88*	I²=0%
Di Bona et al. (15)	2010	grass pollen	19		1518	SLIT	1453	placebo	SS	SMD=-0.32	–0.44~–0.21	*p<0.0001*	I²=55.8%
			17		1430		1358		MS	SMD=-0.33	-0.50~-0.16	*p<0.0001*	I²=78.5%
Li et al. (16)	2018	HDM	5	Adults only		SLIT		placebo	SS	SMD=-0.33	-0.54~-0.13	*p=0.001*	I²=74%
Aini et al. (17)	2021		3		36	ILIT	33	placebo	SS	SMD=-0.27	-0.91~0.38	*p=0.420*	I²=43%
			2		25		23		MS	SMD=-6.56	-21.48~8.37	*p=0.390*	I²=97%
Kim et al. (18)	2021	HDM	10		2803	SLIT-T	2941	placebo	SS	SMD=-0.329	-0.43~-0.23	*p<0.01*	I²=64%
			9		2793		2932		MS	SMD=-0.227	-0.37~-0.08	*p<0.01*	I²=85%
			12		403	SLIT-D	363		SS	SMD=-0.461	-0.80~-0.13	*p<0.01*	I²=77%
			7		181		171		MS	SMD=-0.546	-0.86~-0.23	*p=0.08*	I²=47%
			6		125	SCIT	108		SS	SMD=-1.669	-2.75~-0.59	*p<0.01*	I²=91%
			4		79		66		MS	SMD=-0.697	-1.04~-0.36	*p=0.390*	I²=0%
			26		3331	AIT	3412		SS	SMD=-0.543	-0.69~-0.40	*p<0.01*	I²=83%
			18		3053		3169		MS	SMD=-0.347	-0.48~-0.22	*p<0.01*	I²=76%
Compalati et al. (19)	2009	HDM	8		194	SLIT	188	placebo	SS	SMD=-0.95	-1.77~-0.14	*p=0.02*	I²=92%
			4		89		86		MS	SMD=-1.88	-3.65~-0.12	*p=0.04*	I²=95%
			5	pediatric population	120		115		SS	SMD=-0.49	-1.35~0.37	*p=0.27*	I²=88%
			2		25		22		MS	SMD=-2.43	-6.71~1.84	*p=0.26*	I²=96%
			3	Adult population	74		73		SS	SMD=-1.79	-3.89~0.31	*p=0.09*	I²=95%
			2		64		64		MS	SMD=-1.41	-3.72~0.89	*p=0.23*	I²=97%
Yang et al. (20)	2023		23	<18	2332	SLIT	2380	non-SLIT	SS	SMD=-0.99	-1.29~-0.69	*p<0.001*	I²=95.1%
			17		1980		1902		MS	SMD=-0.78	-1.09~-0.48	*p<0.001*	I²=94.4%
			19		2113		2025		SS	SMD=-1.07	-1.41~-0.74	*p<0.001*	I²=95.5%
			14		1798		1731		MS	SMD=-0.55	-0.82~-0.27	*p<0.001*	I²=92.1%
		grass pollen	10		1544		1451		SS	SMD=-0.32	-0.46~-0.19	*p<0.001*	I²=62.0%
		grass pollen	9		1450		1371		MS	SMD=-0.22	-0.36~-0.08	*p=0.002*	I²=61.7%
		HDM	12		698		848		SS	SMD=-2.11	-2.86~-1.36	*p<0.001*	I²=97.2%
		HDM	7		440		450		MS	SMD=-1.64	-2.73~-0.55	*p=0.003*	I²=97.4%
			5		1279	SCIT	1223	non-SCIT	SS	SMD=-2.52	-3.59~-1.46	*p<0.001*	I²=95.9%
			1		19		17		SS	SMD=-0.54	-1.21~0.13	*p=0.112*	
			3		1154		1119		MS	SMD=-1.42	-3.20~0.36	*p=0.119*	I²=96.9%
		grass pollen	1		12		10		SS	SMD=-0.91	-1.80~-0.03	*p=0.044*	
		grass pollen	1		12		10		MS	SMD=-0.97	-1.86~-0.08	*p=0.032*	
		HDM	4		1267		1213		SS	SMD=-2.94	-4.18~-1.70	*p<0.001*	I²=96.7%
		HDM	2		1142		1109		MS	SMD=-1.62	-3.99~0.75	*p=0.180*	I²=98.0%
Wilson et al. (21)	2005		21		484	SLIT	475	placebo	SS	SMD=–0.42	-0.69~-0.15	*p=0.002*	Chi-square=75.38
			17		405		398		MS	SMD=-0.43	-0.63~-0.23	*p=0.00008*	Chi-square=28,48
		HDM	6		118		110		SS	SMD=-0.58	-1.43~0.27	*p=0.18*	
			3		59		54		MS	SMD=-0.85	-1.93~0.23	*p=0.1*	
		grass pollen	4		144		143		SS	SMD=-0.37	-0.74~0	*p=0.05*	
			4		144		143		MS	SMD=-0.41	-0.81~-0.01	*p=0.04*	
			5	children only	111		107		SS	SMD=-0.31	-1.32~0.7	*p=0.5*	Chi-square=47.16
			3		62		60		MS	SMD=0.02	-0.34~0.37	*p=0.9*	Chi-square=0.43
			16	adults only	373		368		SS	SMD=-0.4	-0.61~-0.18	*p=0.0003*	Chi-square=28.17
			14		343		338		MS	SMD=-0.51	-0.73~-0.29	*p<0.00001*	Chi-square=22.52
Dhami et al. (22)	2017		16		602	SCIT	464	placebo	MS	SMD=-0.52	-0.75~-0.29	*p<0.0001*	I²=64%
			16		632		499		SS	SMD=-0.65	-0.86~-0.43	*p<0.0001*	I²=62%
			58		2978	AIT	2746		SS	SMD=-0.53	-0.63~-0.42	*p<0.0001*	I²=63%
			12	<18	541		547		SS	SMD=-0.25	-0.46~-0.05	*p<0.015*	I²=54%
			23	>18	1557		1406		SS	SMD=-0.56	-0.70~-0.42	*p<0.0001*	I²=62%
			45		2098		1854		MS	SMD=-0.38	-0.49~-0.26	*p<0.0001*	I²=60%
			41		2285	SLIT	2187		SS	SMD=-0.48	-0.61~-0.36	*p<0.0001*	I²=69%
			29		1496		1390		MS	SMD=-0.31	-0.44~-0.18	*p<0.0001*	I²=57%
Zhu et al. (23)	2022		4		64	SCIT	70	placebo	SS	SMD=-2.08	-3.68~-0.48	*p=0.01*	I²=98%
			4		64		70		MS	SMD=-1.43	-2.65~-0.21	*p=0.02*	I²=98%
Feng, B. et al. (24)	2017		26	children only	1147	SLIT	1065	placebo	SS	SMD=-0.55	-0.86~-0.25	*p=0.0003*	I²=90%
			19		814		741		MS	SMD=-0.67	-0.96~-0.38	*p<0.00001*	I²=83%
		HDM	12						SS	SMD=-0.70	-1.43~0.03	*p=0.06*	
			7						MS	SMD=-1.66	-2.60~-0.71	*p=0.0006*	
		grass pollen	14						SS	SMD=-0.43	-0.69~-0.17	*p=0.001*	
			12						MS	SMD=-0.26	-0.44~-0.08	*p=0.005*	
Hoang et al. (25)	2021		4	adults only	77	SCIT	76	placebo	SS	SMD=-2.59	-3.88~-1.29	*p<0.01*	I²=88%
			4		77		76		MS	SMD=-1.84	-3.10~-0.57	*p<0.01*	I²=92%
Radulovic et al. (26)	2011		49		2333	SLIT	2256	placebo	SS	SMD=-0.49	-0.64~-0.34	*p<0.00001*	I²=81%
			38		1737		1642		MS	SMD=-0.32	-0.43~-0.21	*p<0.00001*	I²=50%
		HDM	9		232		232		SS	SND=-0.97	-1.8~-0.3	*p=0.02*	
			5		95		94		MS	SMD=-0.52	-1.09~-0.03	*p=0.07*	
		grass pollen	23		1549		1464		SS	SMD=-0.35	-0.45~-0.24	*p<0.00001*	
			17		1201		1107		MS	SMD=-0.23	-0.37~-0.1	*p=0.0008*	
			34	adults only	1631		1566		SS	SMD=-0.44	-0.56~-0.31	*p=0.0001*	
			26		1168		1067		MS	SMD=-0.4	-0.53~-0.26	*p<0.00001*	
			15	children only	702		690		SS	SMD=-0.52	-0.94~-0.1	*p=0.02*	
			12		569		575		MS	SMD=-0.16	-0.32~0	*p=0.06*	
Calderon et al. (27)	2007		15		597	SCIT	466	placebo	SS	SMD=-0.73	-0.97~-0.50	*p<0.00001*	
			13		549		414		MS	SMD=-0.57	-0.82~-0.33	*p<0.00001*	
Kasemsuk et al. (28)	2022		11		147	LNIT	151	placebo	SS	SMD=-1.37	-2.04~-0.69	*p<0.0001*	I²=84%
			11		139		142		MS	SMD=-1.09	-1.35~-0.83	*p<0.00001*	I²=0%

### 3.3 Quality evaluation

The methodological quality was evaluated using AMSTAR 2. Among the 16 included articles, 5 were rated as “low”, and all the remaining ones were rated as “very low”. None of the articles presented a list of excluded literature and explained the reasons for exclusion. The majority of the studies had not been registered, published, or submitted to the research office or the ethics committee for review. Additionally, in some articles, researchers failed to provide a plausible explanation or discussion of the heterogeneity of findings. Some articles conducted quantitative analyses without a reasonable analysis of publication bias and a discussion of its possible impact on the results. Specific assessment details are provided in **Table 3**.

**Table 3:** Results of the AMSTAR 2 assessment.

References	Q1	Q2	Q3	Q4	Q5	Q6	Q7	Q8	Q9	Q10	Q11	Q12	Q13	Q14	Q15	Q16	Overall quality
Feng, B. H. et al. (13)	Y	N	Y	PY	Y	Y	N	PY	PY	N	Y	N	Y	N	Y	Y	CL
Feng, S. Y. et al. (14)	Y	N	Y	Y	Y	Y	N	Y	PY	N	Y	Y	Y	N	Y	N	CL
Di Bona et al. (15)	Y	N	Y	N	N	N	N	PY	PY	N	Y	Y	Y	Y	Y	Y	CL
Li et al. (16)	Y	N	Y	Y	Y	Y	N	PY	PY	N	Y	Y	Y	Y	N	Y	CL
Aini et al. (17)	Y	Y	Y	Y	Y	Y	N	Y	Y	N	Y	Y	Y	Y	N	Y	CL
Kim et al. (18)	Y	N	Y	Y	Y	Y	N	PY	Y	N	Y	Y	Y	Y	N	N	CL
Compalati et al. (19)	Y	N	Y	Y	Y	Y	N	PY	PY	N	Y	Y	Y	Y	N	N	CL
Yang et al. (20)	Y	Y	Y	Y	Y	Y	N	PY	Y	N	Y	Y	Y	Y	Y	Y	L
Wilson et al. (21)	Y	N	Y	Y	Y	Y	N	PY	PY	N	Y	Y	Y	Y	N	N	CL
Dhami et al. (22)	Y	Y	Y	Y	Y	Y	N	Y	Y	N	Y	Y	Y	Y	Y	Y	L
Zhu et al. (23)	Y	N	Y	Y	Y	Y	N	PY	Y	N	Y	Y	Y	Y	N	Y	CL
Feng, B. et al. (24)	Y	N	Y	Y	N	N	N	PY	Y	N	Y	Y	Y	N	Y	N	CL
Hoang et al. (25)	Y	Y	Y	Y	Y	Y	N	PY	Y	N	Y	Y	Y	Y	Y	Y	L
Radulovic et al. (26)	Y	PY	Y	Y	Y	N	N	PY	Y	N	Y	Y	Y	Y	N	Y	CL
Calderon et al (27).	Y	PY	Y	Y	Y	Y	N	PY	Y	N	Y	Y	Y	Y	Y	N	L
Kasemsuk et al. (28)	Y	Y	Y	Y	Y	Y	N	PY	Y	N	Y	Y	Y	Y	Y	Y	L

Y, yes; PY, partial yes; N, no; CL, critically low; L, low; M, moderate; H, high.

The quality of the evidence was evaluated by using the GRADE system. It was discovered that the quality of evidence in most studies was not satisfactory. Among the included articles, six had evidence of moderate quality, six had low-quality evidence, and four had very low-quality evidence, while no high-quality evidence was found. Inconsistency was the most common factor for downgrading across programs, possibly due to the differences in the populations included in the studies, allergens, or interventions. None were downgraded because of Indirectness. Specific details of the assessments are provided in **Table 4**.

**Table 4:** Assessments of the GRADE.

References	Risk of bias	Inconsistency	Indirectness	Imprecision	Publication bias	Evidence quality
Feng, B. H. et al. (13)	0	-1	0	0	-1	L
Feng, S. Y. et al. (14)	0	-1	0	0	0	M
Di Bona et al. (15)	0	-1	0	0	0	M
Li et al. (16)	0	-1	0	0	0	M
Aini et al. (17)	0	-1	0	0	-1	L
Kim et al. (18)	0	-1	0	0	-1	L
Compalati et al. (19)	0	-1	0	0	-1	L
Yang et al. (20)	0	-1	0	0	0	M
Wilson et al. (21)	0	-2	0	0	-1	VL
Dhami et al. (22)	0	-1	0	0	0	M
Zhu et al. (23)	-1	-1	0	0	-1	VL
Feng, B. et al. (24)	-1	-1	0	0	-1	VL
Hoang et al. (25)	0	-1	0	-1	0	L
Radulovic et al. (26)	-1	-1	0	0	-1	VL
Calderon et al (27).	0	-1	0	0	0	M
Kasemsuk et al. (28)	0	-1	0	-1	0	L

VL, very low; L, low; M, moderate; H, high.

**Figure 2:**
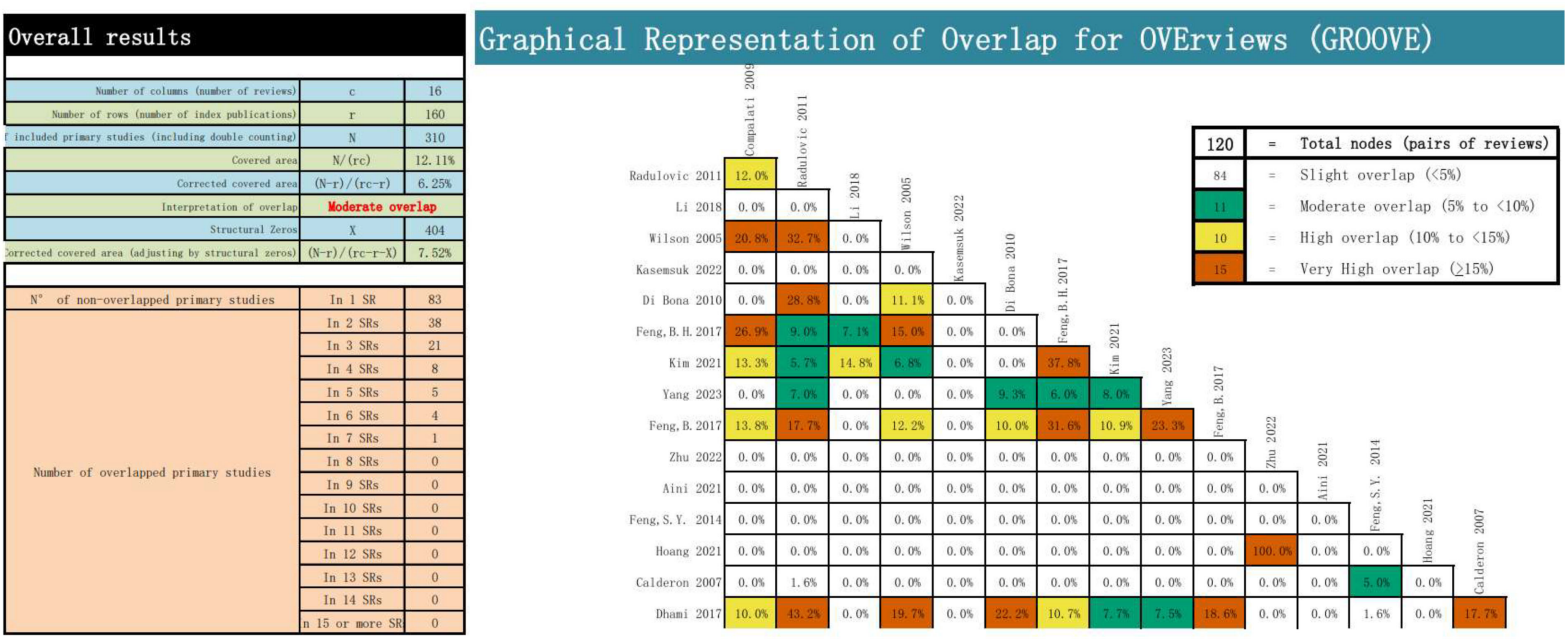
Overlapping of the included reviews.

We employed the GROOVE tool to evaluate the overlap of the main studies in the included literature. It was discovered that there was a moderate overlap among the included articles. The tool calculates the overlap rate by using the formula (N-r)/(rc-r). There were 120 nodes among the included articles, of which 84 nodes had a slight overlap, 11 nodes had a moderate overlap, 10 nodes had a high overlap, and 15 nodes had a very high overlap. The overall overlap was moderate, reaching 6.25%. The detailed evaluation results are presented in **Figure 2**.

### 3.4 Evaluation of the efficacy outcome

Due to the diverse allergens of AR patients in the included literature, the varied interventions, and the distinct efficacies in adults and children, a subgroup analysis was conducted.

#### 3.4.1 The efficacy of SLIT

**Figure 3:**
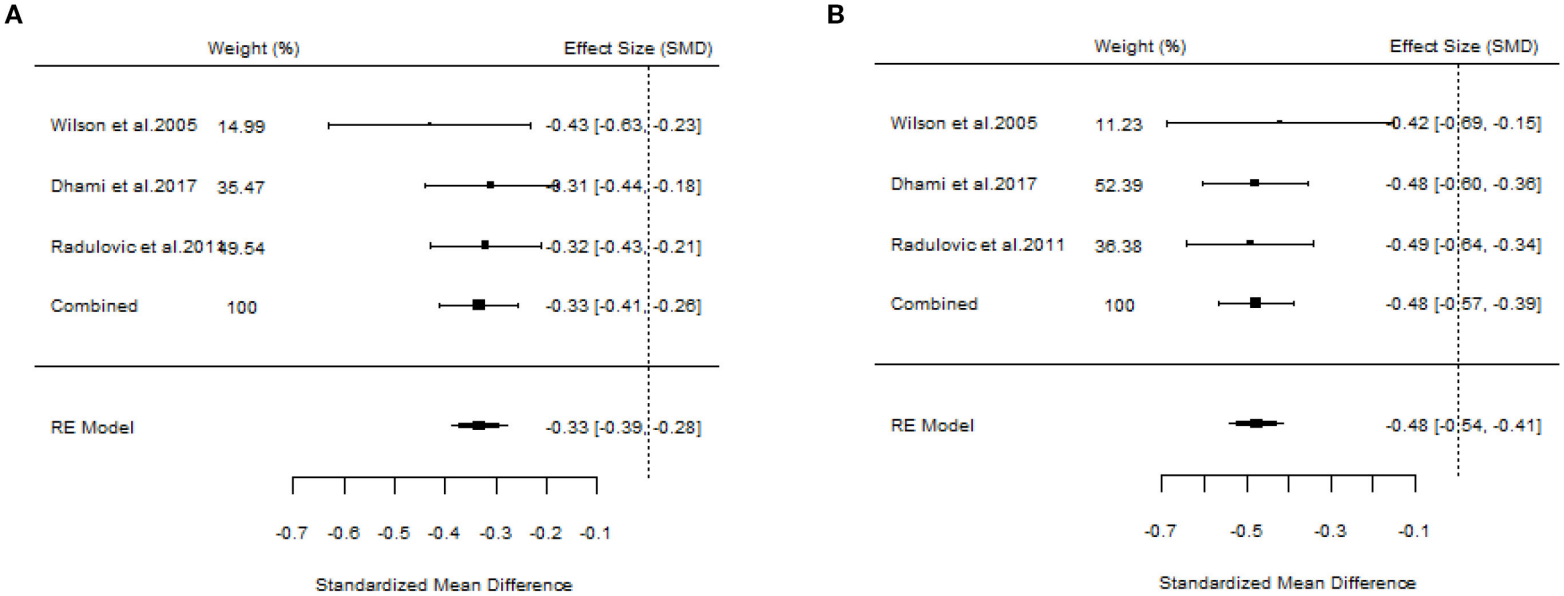
Forest plot of SLIT efficacy in a population without allergen or age discrimination. [**(A)** Symptom scores. **(B)** Medication scores].

**Figure 4:**
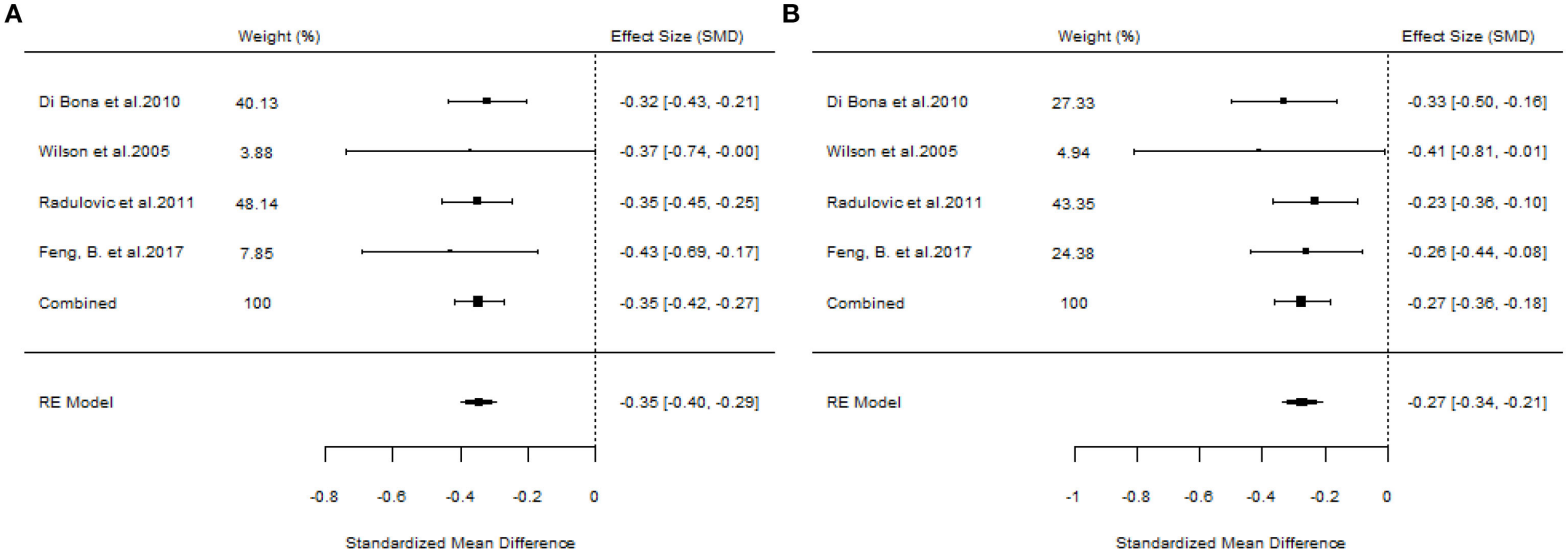
Forest plot of the efficacy of SLIT in AR patients allergic to grass pollen. [**(A)** Symptom scores. **(B)** Medication scores].

**Figure 5:**
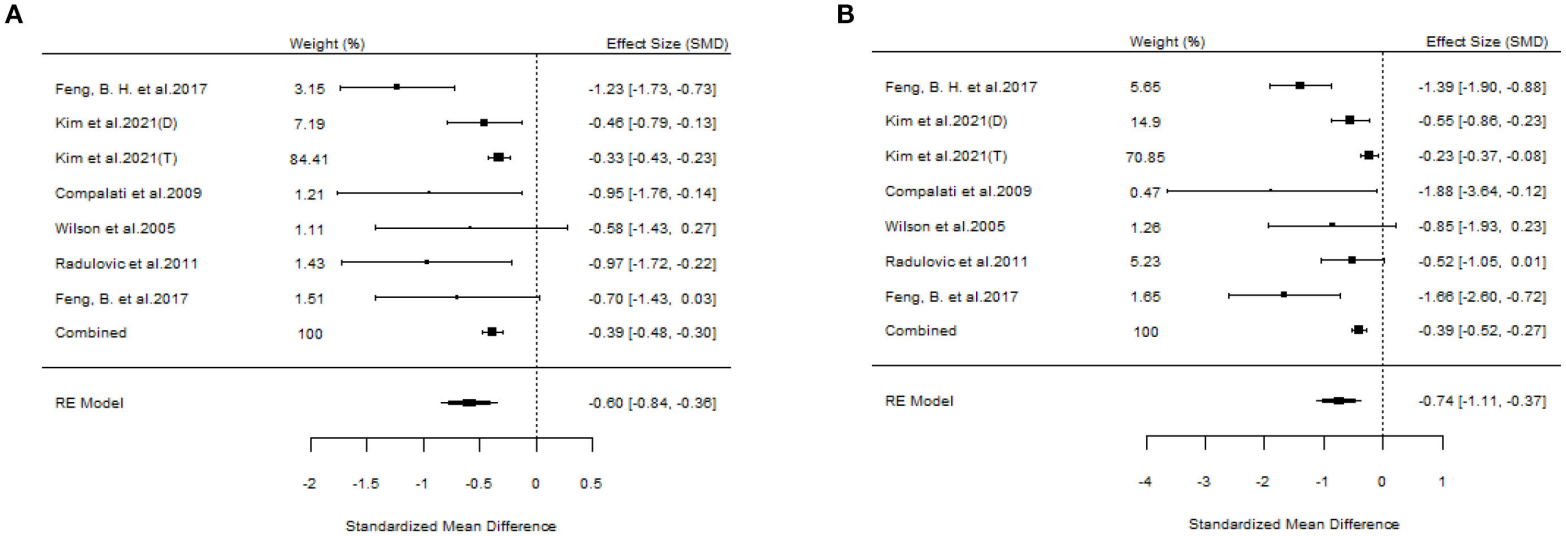
Forest plot of the efficacy of SLIT in AR patients allergic to HDM. [**(A)** Symptom scores. **(B)** Medication scores].

**Figure 6:**
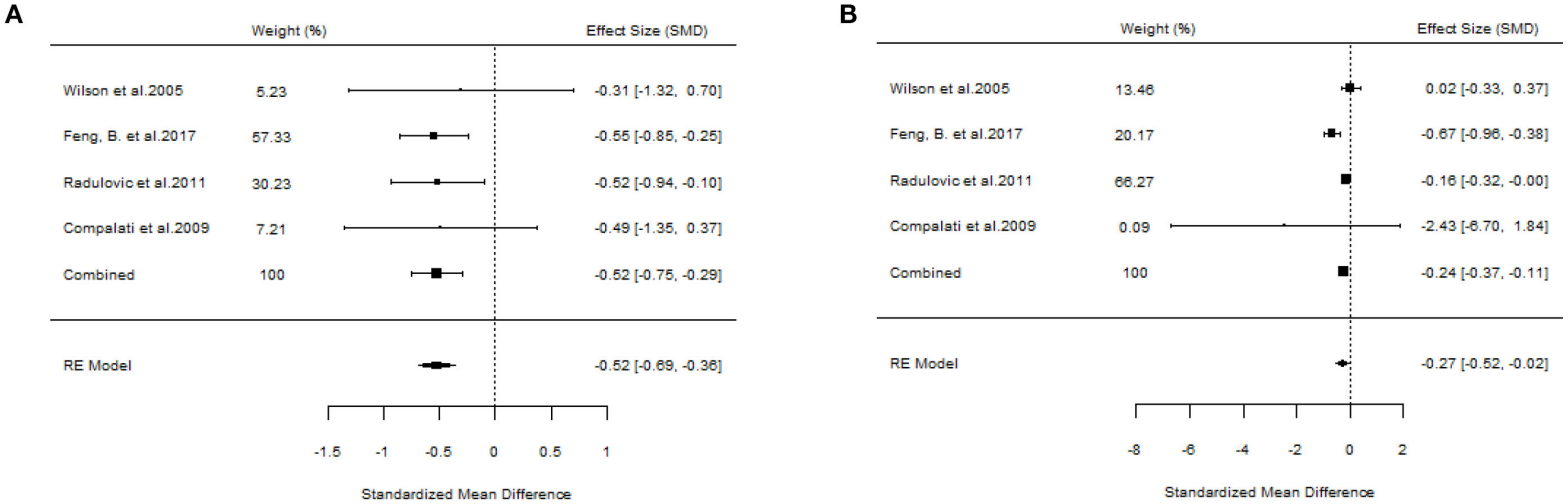
Forest plot of the efficacy of SLIT in children with AR. [**(A)** Symptom scores. **(B)** Medication scores].

**Figure 7:**
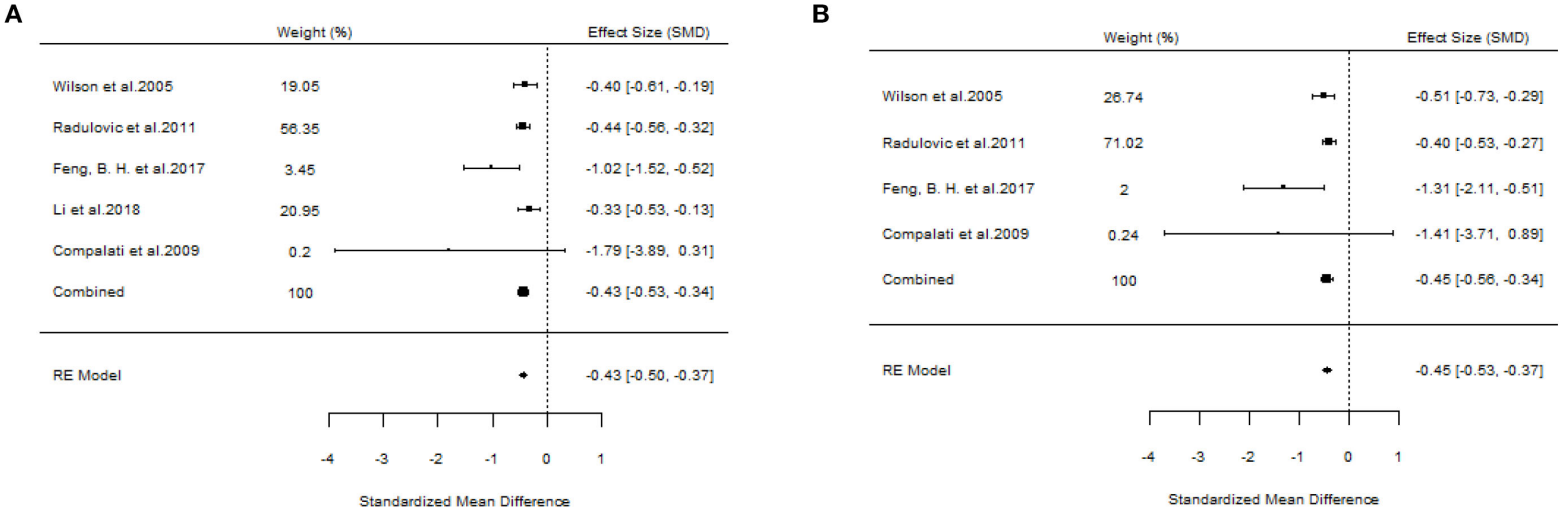
Forest plot of the efficacy of SLIT in adult AR patients. [**(A)** Symptom scores. **(B)** Medication scores].

A total of 10 articles dealt with the efficacy of SLIT. Among them, three studies failed to distinguish the age and allergen of the participants (21, 22, 26). All these three studies provided positive outcomes, demonstrating that SLIT reduced symptom scores and medication scores in AR patients compared with placebo. We extracted the 95%CI reported by each study to assess the overall efficacy. The results indicated that SLIT was effective in the population without discrimination of allergen and age (SS: SMD=-0.48, 95%CI=-0.57~-0.39, *p* = 2.41e-48, I²=0%, Egger’s test: *p* = 0.77; MS: SMD=-0.33, 95%CI=-0.41~-0.26, *p* = 9.38e-33, I²=0%, Egger’s test: *p* = 0.55) (**Figure 3**). Five studies identified grass pollen as the allergen in patients with AR (15, 20, 21, 24, 26). As the study by Yang et al. focused on comparing SLIT with non-SLIT treatments, we extracted the 95% confidence intervals from the remaining four studies to assess the overall efficacy. The results indicated that SLIT could significantly reduce both symptom scores and medication scores in AR patients sensitized to grass pollen (SS: SMD=-0.35, 95%CI=-0.42~-0.27, *p* = 2.30e-39, I²=0%, Egger’s test: *p* = 0.71; MS: SMD=-0.27, 95%CI=-0.36~-0.18, *p* = 1.46e-17, I²=0%, Egger’s test: *p* = 0.55) (**Figure 4**). Seven studies included patients with AR sensitized to HDM allergens (13, 18–21, 24, 26). After excluding the study by Yang et al., in which the control group received a different intervention, we assessed the overall efficacy of SLIT in the remaining studies. The results indicated that SLIT was effective in treating AR patients with HDM allergy (SS: SMD=-0.39, 95%CI=-0.48~-0.30, *p* = 1.46e-06, I²=83.86%, Egger’s test: *p* = 0.0012; MS: SMD=-0.39, 95%CI=-0.52~-0.27, *p* = 9.78e-05, I²=89.79%, Egger’s test: *p* = 0.0006) (**Figure 5**). Our study results revealed high heterogeneity and Egger’s test P < 0.05, indicating bias in this study. Five articles provided study results in pediatric patients (19–21, 24, 26). Although the majority of the findings in these included studies did not reach statistical significance, the overall efficacy assessment indicated that SLIT demonstrated effectiveness in pediatric AR patients (SS: SMD=-0.52, 95%CI=-0.75~-0.29, *p* = 3.17e-10, I²=0%, Egger’s test: *p* = 0.74; MS: SMD=-0.24, 95%CI=-0.37~-0.11, *p* = 0.0356, I²=78.68%, Egger’s test: *p* = 0.34) (**Figure 6**). We suspected that either the sample size of the studies included in the original meta-analysis was too small or there was bias in some of the studies, resulting in insignificant results. There were also five studies evaluating the efficacy of SLIT in adults (13, 16, 19, 21, 26). Among these, the study conducted by Li et al. reported only the symptom score results. Based on our comprehensive assessment, we found that SLIT is also effective in treating adult patients with AR (SS: SMD=-0.43, 95%CI=-0.53~-0.34, *p* = 2.69e-37, I²=0.0093%, Egger’s test: *p* = 0.0781; MS: SMD=-0.45, 95%CI=-0.56~-0.34, *p* = 5.62e-28, I²=0%, Egger’s test: *p* = 0.0398) (**Figure 7**).

#### 3.4.2 The efficacy of SCIT

**Figure 8:**
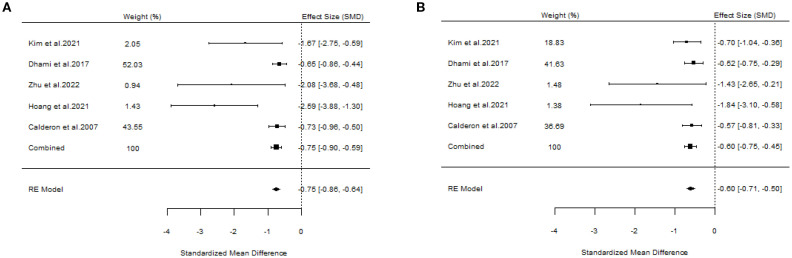
Forest plots of SCIT efficacy in AR patients. [**(A)** Symptom scores. **(B)** Medication scores].

A total of six studies evaluated the efficacy of SCIT (18, 20, 22, 23, 25, 27). A pooled analysis of all studies meeting the inclusion criteria for overall assessment indicated that the overall findings were valid (SS: SMD=-0.75, 95%CI=-0.90~-0.59, *p* = 1.17e-40, I²=0%, Egger’s test: *p* = 0.0007; MS: SMD=-0.60, 95%CI=-0.75~-0.45, *p* = 1.96e-29, I²=0.031%, Egger’s test: *p* = 0.0327) (**Figure 8**). The results of our study demonstrated little heterogeneity, but Egger’s test indicated that the study was biased at *p* < 0.05.

#### 3.4.3 The efficacy of other interventions

**Figure 9:**
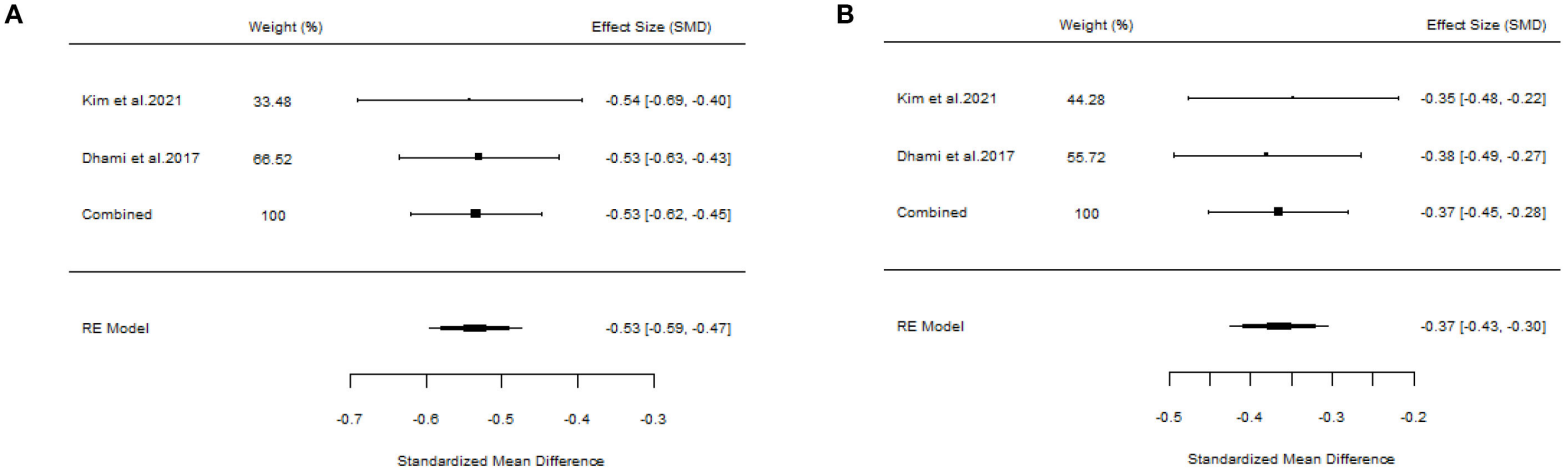
Forest plot of the efficacy of AIT in AR patients. [**(A)** Symptom scores. **(B)** Medication scores].

Two studies failed to present specific AIT methods (18, 22), and the outcomes were positive. We also assessed the overall results (SS: SMD=-0.53, 95%CI=-0.62~-0.45, *p* = 5.07e-67, I²=0%, Egger’s test: *p* = 0.9275; MS: SMD=-0.37, 95%CI=-0.45~-0.28, *p* = 3.98e-32, I²=0%, Egger’s test: *p* = 0.9201) (**Figure 9**). Additionally, there was another article concerning cluster SCIT (14) (SS: WMD=-5.91, 95%CI=-13.68~1.87, *p* = 0.14, I²=89%; MS: WMD=-1.27, 95%CI=-2.83~0.29, *p* = 0.11, I²=94%), suggesting that cluster SCIT did not achieve superior efficacy compared with placebo in the treatment of AR. Aini et al. (17), a meta-analysis of ILIT, also produced negative results (SS: SMD=-0.27, 95%CI=-0.91~0.38, *p* = 0.42, I²=43%; MS: SMD=-6.56, 95%CI=-21.48~8.37, *p* = 0.39, I²=97%). The meta-analysis by Kasemsuk et al. (28) on LNIT demonstrated positive results (SS: SMD=-1.37, 95%CI=-2.04~-0.69, p<0.0001, I²=84%; MS: SMD=-1.09, 95%CI=-1.35~-0.83, *p* < 0.00001, I²=0%).

#### 3.4.4 Impact on asthma progression

The included studies have demonstrated that AIT exhibits varying degrees of efficacy in the prevention and control of asthma among patients with AR, with differences in both effectiveness and safety across different administration routes. Among the available treatment options, SCIT has the strongest supporting evidence. According to Calderon et al. (27), SCIT significantly reduced bronchial symptom scores in patients with AR and asthma (SMD=-0.59, 95%CI=-1.06~-0.11, *p* = 0.02). Furthermore, SCIT can reduce the need for rescue medications (e.g., inhaled β_2_ agonists), thereby contributing to improved lung function, as indicated by increased forced expiratory volume in one second (FEV_1_) and decreased peak expiratory flow (PEF) variability. The therapeutic benefits of SCIT can be sustained for 3 to 5 years following the completion of a long-term treatment regimen (≥3 years). Additionally, the study highlights allergic rhinitis as a well-established risk factor for asthma, with 15% to 38% of individuals diagnosed with AR also developing asthma.

SLIT offers advantages in terms of safety and applicability for pediatric populations. Compalati et al. (19) demonstrated that SLIT could significantly reduce bronchial symptom scores in dust mite-allergic AR patients with asthma (SMD=-0.95, 95%CI=-1.74~-0.15, *p* < 0.05). In the subgroup of children, the reduction in symptoms was even more pronounced (SMD=-1.09, 95%CI=-1.96~-0.22, *p* = 0.01), accompanied by a significant decrease in asthma medication use (SMD=-1.48, 95%CI=-2.70~-0.26, *p* = 0.02). However, a high level of heterogeneity was observed (I (2) = 93%~96%), which may be attributed to variations in allergen dosage and treatment duration. Dhami et al. further confirmed that SLIT significantly improved both asthma symptoms (SMD=-0.49, 95%CI=-0.69~-0.30) and medication use (SMD=-0.37, 95%CI=-0.69~-0.30) in pollen-allergic AR patients with asthma. Moreover, symptom improvement was sustained for at least one year after discontinuation of treatment. A network meta-analysis by Kim et al. (18) indicated no significant difference between SLIT and SCIT in terms of asthma symptom control among pollen-allergic AR patients (*p* = 0.43). However, SLIT was associated with a lower incidence of local adverse reactions, such as oral pruritus (<5% vs. 10%~15%).

ILIT represents a novel approach to AIT. According to Aini et al. (17), ILIT demonstrated a significantly improved safety profile, with a reduced incidence of cutaneous reactions, such as localized swelling, compared to SCIT (RR = 0.31, 95%CI=0.13~0.72, *p* = 0.007). Furthermore, ILIT was associated with a more rapid decline in serum-specific IgE levels (3 months vs. 3 years). However, no statistically significant differences were observed between ILIT and placebo in terms of asthma symptom scores (SMD=-0.27, 95%CI=-0.91~0.38, *p* = 0.42) or medication usage. Given the high heterogeneity among trials (e.g., variations in allergen dosages and injection intervals) and the limited evidence supporting the efficacy of ILIT in modifying the course of asthma, ILIT is not recommended as the first-line allergen immunotherapy for patients with allergic rhinitis and asthma.

## 4 Discussion

Allergic rhinitis is a highly common chronic disease, and its progression is also associated with asthma, sinusitis, and other disorders (29). Its pathogenesis is a non-infectious inflammatory response driven by helper T lymphocyte 2 (Th2) in atopic individuals upon inhalation of allergens (30). The occurrence of allergy might be related to the spread of antigenic determinants, and the mechanism of desensitization treatment is to reduce the spread of this determinant, continuously apply allergens to establish peripheral specific non-reactive T cells, and inhibit cytokine activity and T cell proliferation (31). Although AIT has been practiced for over a hundred years and is the sole treatment targeting the underlying pathophysiology and altering the natural course of AR, details regarding the practice of AIT during its evolution have varied globally. Since the 1980s, several national and regional allergy societies have endeavored to provide some guidance on AIT practice. However, it was not until 2009 that the World Allergy Organization (WAO) issued the consensus on SLIT for the first time. Up to now, no such global consensus document exists for other types of immunotherapy (32).

In recent years, the number of SRs/MAs regarding the application of AIT in the treatment of AR has increased. However, due to variations in evaluation systems, the quality of these studies is inconsistent, leading to suboptimal outcomes. This study introduces an innovative approach by shifting the core variable in treatment decision-making from “target/allergen” to “route of administration,” thereby enabling the direct translation of evidence into actionable clinical decisions. We conducted a detailed and comprehensive review of 16 studies. The findings indicated that the majority of the included SRs/MAs reported that various approaches of AIT in the treatment of AR were effective, particularly SLIT and SCIT. However, some studies still suggested that the efficacy of AIT in treating AR remained inconclusive, especially in pediatric populations and studies focusing on cluster SCIT and ILIT. We hypothesize that this inconsistency may be attributable to the limited or biased sample sizes of the included SRs/MAs. Additionally, the less pronounced therapeutic effects observed in children compared to adults may be associated with lower treatment compliance among pediatric patients.

Some other studies have included safety analyses. Feng, S.Y. and Calderon et al. discovered that the utilization of AIT might lead to side effects such as rhinoconjunctivitis, mild wheezing, urticaria, ear itching, palm/sole itching, and eyelid edema. The research of Radulovic and Yang et al. indicated that in comparison with the subcutaneous approach, the application of AIT could cause adverse effects like nasal conjunctivitis, mild wheezing, urticaria, ear itching, palm/sole itching, and eyelid edema. The sublingual route is safer and has fewer side effects, which is in line with previous studies (33). Compared to dosage forms, Li et al. found that SLIT tablets could better regulate the drug volume and achieve higher compliance than SLIT drops due to their safety and ease of transport, administration, and follow-up.

In addition to comparing the relative efficacy of different administration routes of AIT in the treatment of AR, this study also examined the potential long-term outcomes associated with allergic progression. Although the reviewed literature did not provide a systematic summary of the direct effects of AIT on the incidence of new-onset asthma or its progression in patients with AR, several studies suggest that AIT administered via various routes may have a beneficial impact on the prevention and alleviation of asthma. These findings hold clinical significance. In recent years, real-world studies have demonstrated that AIT, including both subcutaneous and sublingual administration—available in either liquid or tablet form—is effective for patients with AR, regardless of whether they have asthma or not. SCIT can effectively reduce symptom scores and the need for rescue medication in patients with moderate to severe stable asthma who suffer from HDM or pollen allergies. Furthermore, SCIT can decrease the frequency of acute exacerbations by 40% to 50%, with its long-term therapeutic effects lasting for 3 to 5 years (34, 35). SLIT liquid significantly reduced symptom scores (SMD=-0.30) and medication scores (SMD=-0.51) across 25 RCTs involving 1,830 cases. According to the French national study, SLIT liquid decreased the risk of asthma exacerbation and escalation of GINA treatment steps. Moreover, the reduction in the risk of new-onset asthma among children was particularly pronounced (HR = 0.51) (36, 37). SLIT tablets demonstrated efficacy in improving allergic rhinitis symptoms triggered by grass or birch pollen, particularly in patients with mild asthma (SMD=-0.36), as well as in reducing medication use (SMD=-0.29). The therapeutic effect was sustained even after a two-year period of treatment discontinuation. After six years of follow-up, the rate of asthma medication withdrawal was significantly higher in the SLIT group (49.1%) compared to the control group (35.1%) (38, 39). Children represent a key population for AIT in the prevention of asthma, while the benefits of AIT in adults primarily focus on disease control and delaying the progression of asthma. Despite encouraging evidence from real-world studies, higher-quality research is needed to clarify the role of different administration routes in the context of new-onset asthma. Future studies should incorporate new-onset asthma as a key outcome, with a standardized definition and systematic evaluation within prospective cohort studies.

A major advantage of this study lies in its utilization of an umbrella review to reevaluate the existing evidence and synthesize higher-level evidence. It is beneficial for clinicians to determine whether to select different AIT to treat AR with various allergens in different populations. Nevertheless, the study has certain limitations: (1) According to the AMSTAR 2 method, no high-quality studies were included; most studies were not registered, and most systematic reviews and meta-analyses failed to consider the bias risk of the included literature and the heterogeneity of the study results. (2) The assessment of methodological quality and evidence quality is subjective. Even if we conduct a detailed and objective evaluation of each item of the evaluation system, guidelines or authoritative third parties are needed to adjudicate disputes. There might still be some variations in the results. (3) Only studies published in English were incorporated, which might have a certain bias risk. (4) The main outcome index of the RCTs included in this study was the scoring scale, and there might be some differences in the content of the RCTs, which could have an impact on the treatment outcome and needs to be verified by objective indicators such as serum specific IgE level. (5) Both ILIT and cluster SCIT were each covered by only one study and were found to be ineffective. Due to the high heterogeneity among trials and the small sample sizes, future trials should involve more participants and report standardized management and outcome measures of the study. Given the limitations of this study, further high-quality research is necessary.

## 5 Conclusion

In conclusion, both SLIT and SCIT are effective treatments for AR, showing significant efficacy in adults, children, and for various allergens. SLIT is relatively safe and easy to comply with. However, meta-analyses and systematic reviews of cluster SCIT, ILIT, and LNIT are limited, and their efficacy evaluation needs improvement. Additionally, the methodological quality and evidence of existing systematic reviews and meta-analyses are generally low, so these results should be interpreted cautiously. Therefore, more high-quality, large-scale, multicenter, randomized controlled trials are needed to firmly validate the efficacy of different AIT methods for treating AR across various populations and allergens.

## Data Availability

The original contributions presented in the study are included in the article/supplementary material. Further inquiries can be directed to the corresponding author.
